# Parental rejection in early adolescence predicts a persistent ADHD symptom trajectory across adolescence

**DOI:** 10.1007/s00787-021-01844-0

**Published:** 2021-07-17

**Authors:** Djûke M. Brinksma, Pieter J. Hoekstra, Annelies de Bildt, Jan K. Buitelaar, Barbara J. van den Hoofdakker, Catharina A. Hartman, Andrea Dietrich

**Affiliations:** 1 Department of Child and Adolescent Psychiatry, University Medical Center Groningen, University of Groningen, Lübeckweg 2, NL-9723 HE Groningen, The Netherlands; 2 Department of Cognitive Neuroscience, Donders Institute for Brain, Cognition and Behavior, Nijmegen, The Netherlands; 3 Karakter Child and Adolescent Psychiatry University Centre, Nijmegen, The Netherlands; 4 Department of Psychiatry, University Medical Center Groningen, University of Groningen, Interdisciplinary Center Psychopathology and Emotion Regulation (ICPE), Groningen, The Netherlands

**Keywords:** ADHD, Gene–environment interactions, Parenting style, Longitudinal, Trajectories

## Abstract

Despite a general decrease of attention-deficit/hyperactivity disorder (ADHD) symptoms during adolescence, these may persist in some individuals but not in others. Prior cross-sectional studies have shown that parenting style and their interaction with candidate genes are associated with ADHD symptoms. However, there is a lack of longitudinal research examining the independent and interactive effects of parenting and plasticity genes in predicting the course of attention-deficit/hyperactivity disorder (ADHD) symptoms across adolescence. Here, we investigated how children perceived their parents’ parenting style (i.e., rejection, overprotection, and emotional warmth) at the age of 11, and their interaction with *DRD4,* *MAOA,* and *5-HTTLPR* genotypes on parent-reported ADHD symptoms at three time points (mean ages 11.1, 13.4, and 16.2 years) in 1730 adolescents from the TRacking Adolescents’ Individual Lives Survey (TRAILS). Growth Mixture Modeling in M*plus* identified four ADHD symptom trajectories: low, moderate stable, high decreasing, and high persistent. Perceived parental rejection predicted class membership in the high persistent trajectory compared to the other classes (*p* < 0.001, odds ratios between 2.14 and 3.74). Gene-environment interactions were not significantly related to class membership. Our results indicate a role of perceived parental rejection in the persistence of ADHD symptoms. Perceived parental rejection should, therefore, be taken into consideration during prevention and treatment of ADHD in young adolescents.

## Introduction

Attention-deficit/hyperactivity disorder (ADHD) is a childhood-onset disorder with core symptoms of inattention and hyperactivity/impulsivity, which may persist into adulthood [1]. ADHD symptoms vary along a continuum, with symptoms in the non-clinical range at the lower end and severe symptoms in the clinical range at the upper end [2], which have been suggested to share the same etiology [3]. Although ADHD symptoms tend to decrease during adolescence [4], the course of symptoms differs between individuals. Various symptom trajectories have been described across adolescence, in clinical and population samples, mostly including a low stable and a high persistent trajectory (e.g., [5–7]). Adolescence is a sensitive maturational period during which further development may be shaped [8]. However, still little is known which factors characterize adolescents who remit versus those who have a more persistent course of ADHD symptoms in the transition towards adulthood. Here, we focused on the role of perceived parenting style and ADHD candidate genes on the course of ADHD symptoms across adolescence.

Negative parenting styles have been associated in cross-sectional studies with ADHD symptoms in childhood and adolescence; examples are maternal overprotection and control [9], parental rejection [10], as well as high levels of inconsistent parental discipline and anger, and low levels of parental involvement [11, 12]. Moreover, parental criticism [6], low parental emotional support and intellectual stimulation [13], and inconsistent discipline [14] have been found in relation to trajectories of persistently high ADHD symptom levels from childhood into late adolescence. In contrast, positive parenting (i.e., appropriate parental involvement) has been found to prospectively predict reduced levels of ADHD symptoms at 1-year follow-up in early childhood [15]. Also, two longitudinal studies showed that higher levels of parental warmth were related to reduced rates of ADHD over time [16]. Currently, associations of other parenting styles such as parental rejection and overprotection with ADHD symptom trajectories across adolescence remain unclear. As children’s ADHD symptoms may induce high levels of child-rearing stressors, their parents may display high levels of over-reactivity (possibly perceived as rejection) or tend to be more critical (possibly perceived as overprotection; [17]). A better understanding of the role of children perceiving their parents’ parenting style as negative, such as parental rejection and overprotection or low emotional warmth, as well as of positive parenting, such as high emotional warmth, in relation to the course of ADHD symptoms may help promote youth development. Parental rejection is characterized by hostility, punishment, and blaming. Given a person’s need for warmth and belongingness [18], it has long-time been clear that a family environment characterized by rejection is associated with problem behavior (e.g., [19]) including ADHD symptoms [10]. Overprotection (i.e., fearfulness and anxiety for the child’s safety, guilt engendering, and intrusiveness) is described [9] as a proxy of a poor child–parent relation associated with elevated levels of ADHD symptoms in a cross-sectional study.

Moreover, the influence of parenting styles on ADHD symptoms may depend on the adolescent’s genotype, whereby genes influence sensitivity to both supportive as well as adverse environments [20]. Cross-sectional studies indicated gene–environment (G × E) interactions between the 7-repeat allele of the *dopamine* *D4* *receptor* gene (*DRD4*) and parenting factors (e.g., consistent parenting, sensitive maternal care) in association with ADHD or externalizing symptoms, for better or for worse (i.e., positive parenting in those with the 7-repeat allele was associated with fewer symptoms and negative parenting with more symptoms [21, 22]). Consistent with the differential susceptibility hypothesis [23], a ‘for better and for worse’ mechanism was also found by Janssens and colleagues [24] who reported that adolescents carrying the 7-repeat allele of the *DRD4* showed fewer externalizing symptoms when experiencing high levels of parental proactive control, while adolescents showed more externalizing symptoms when experiencing lower levels of parental proactive compared to adolescents without this variant. Similarly, longitudinal studies showed that individuals carrying the *DRD4* 7-repeat allele had fewer ADHD symptoms in childhood [25] and adolescence [26] when they had experienced higher levels of sensitive and stimulating maternal care in infancy and early childhood, while higher levels of symptoms were found in the face of lower early maternal sensitive care [25, 26]. In relation to parenting and ADHD, the literature also suggests a role for the high-activity *monoamine* *oxidase* *A* (*MAOA*) genotype, i.e., negative parenting predicted inattention symptoms only among boys with high-activity *MAOA* [27]; and for the low-activity *serotonin* *transporter* (*5-HTTLPR*) genotype, i.e., family conflict predicted inattention symptoms only in those with low-activity *5-HTTLPR* [28].

In the present longitudinal study, we investigated whether the way children perceived their parents’ parenting style (as assessed in early adolescence), would predict subsequent ADHD symptom trajectories across adolescence. We used perceived parenting styles (i.e., rejection, overprotection, and emotional warmth) from an existing large pooled population and clinic-referred cohort. The availability of separate raters of parenting style and of the course of ADHD symptoms enabled us to avoid same rater bias, i.e. halo effects [29]. We expected that class membership in unfavorable ADHD symptom trajectories (e.g., high persistent) would be predicted by a higher level of perceived parental rejection and/or overprotection, or a lower level of perceived emotional warmth, whereas we expected a reverse pattern for membership in a more favorable trajectory (e.g., low levels, remitting). As a second aim, we explored the role of the ADHD candidate genes *DRD4,* *MAOA,* and *5-HTTLPR* in interaction with perceived parenting as a predictor of ADHD symptom trajectories.

## Method

### Sample

Our study included 1730 adolescents assessed at 3 time points T1 (*M*_age_ = 11.1, range 10.0‒12.6), T2 (*M*_age_ = 13.4, range 11.6‒15.1), and T3 (*M*_age_ = 16.2, range 14.4‒18.4) as part of the population-based and clinic-referred cohort Tracking Adolescents’ Individual Lives Survey (TRAILS). TRAILS is a prospective cohort study of Dutch adolescents with the aim to chart and explain the development of mental health from early adolescence into adulthood, both at the level of psychopathology and the levels of underlying vulnerability and environmental risk. The population-based cohort comprised young adolescents from five municipalities in the north of the Netherlands, including urban and rural areas. The inclusion of the clinic-referred cohort, which started 2 years later, was based on referral to a child and adolescent psychiatric outpatient clinic in the Northern Netherlands. About 20.8% had been referred at age ≤ 5 years, 66.1% between age 6 and 9 years, and 13.1% between age 10 and 12 years. The sampling procedures, descriptive statistics, and response rates of both cohorts have been well documented elsewhere [30].

At T1, the total sample consisted of 2773 adolescents from the population-based (*n* = 2230) and clinic-referred cohort (*n* = 543), with retention rates for both cohorts over 80% at T2 and T3. For the present study, only participants with complete genetic data on the *DRD4,* *MAOA*, and *5-HTTLPR* genotypes were included (*n* = 1761) in the analyses, that were collected at T2 or T3 of the study, explaining the more limited availability of data. Participants with complete genetic data did not differ in the proportion of males versus females [*χ*^2^ (1) = 0.93, *p* > 0.05], severity of ADHD symptoms at T1 [*t* (2588) = − 1.23, *p* > 0.05], but had a higher socio-economic status [SES; *χ*^2^ (2) = 74.72, *p* < 0.001], and were more likely to be of Dutch ancestry [*χ*^2^ (1) = 33.07, *p* < 0.05] when compared to those without complete genetic data. Subsequently, subjects were excluded if there was no information available at all on perceived parenting (*n* = 10) or ADHD symptoms (*n* = 21). The final sample consisted of 1730 adolescents of which 1364 adolescents (78.8% of the final sample; 89.6% Dutch ancestry) were from the population-based cohort and 366 adolescents (21.2% of the final sample; 98.6% Dutch ancestry) from the clinic-referred cohort.

### Measures

#### ADHD symptoms

At all three waves, the DSM IV-Oriented subscale Attention-Deficit/Hyperactivity Problems of the parent-rated Child Behavioral Checklist [31] consisting of seven items (three inattention and four hyperactivity-impulsivity items) was used to measure ADHD symptoms. Items were scored on a three-point Likert-scale ranging from 0 (‘not true’) to 2 (‘very true or often true’). The DSM-oriented subscale of the CBCL has shown good reliability as well as convergent and discriminative validity in adolescents [32]. The scale’s internal consistency coefficients ranged between 0.82 and 0.85 across the three time points and both cohorts, which can be considered as good.

For descriptive purposes of ADHD symptom trajectories, ASEBA cut-off scores [33] were used to categorize adolescents with clinical (> 97th percentile), subclinical (between 90 and 97th percentile), and normal (< 90th percentile) ADHD symptom levels.

#### Perceived parenting

At T1, adolescent’s current perception of parental rearing were assessed with the short version of the Egna Minnen Beträffande Uppfostran (My Memories of Upbringing) for Children (EMBU-C [34]). The 47 items were scored on a 4-point Likert-scale ranging from 1 (‘no, never’) to 4 (‘yes, almost always’) separately for perceived father and mother rearing style. The *rejection* scale included 12 questions about hostility, punishment, and blaming the child (5 of the originally 17 questions were excluded due to low loadings, see [35]; Cronbach’s *α* = 0.84). The *overprotection* scale comprised 12 items about fearfulness and anxiety for the child’s safety, and intrusiveness (Cronbach’s *α* = 0.90). The 18 items of the *emotional* *warmth* scale refer to giving special attention, praise, and unconditional love (Cronbach’s *α* = 0.94). We computed a single mean score as in previous studies, to increase comparability and to reduce the number of tests (e.g., [24, 36]). Answers for both parents were highly correlated (*r* = 0.81 for rejection, *r* = 0.64 for overprotection, and *r* = 0.77 for emotional warmth).

#### Genotyping

DNA was extracted from blood samples (*n* = 1443) or buccal swabs with a Cytobrush (*n* = 287) and was collected at T2 for the clinic-referred cohort and at T3 for the population-based cohort. Genotyping of the length polymorphisms *DRD4,* *MAOA,* *HTTLPR*, and SNP rs25331 (A/G SNP in *L* *HTTLPR*) was done at the Research lab for Multifactorial Diseases within the Human Genetics department of the Radboud University Nijmegen Medical Centre in Nijmegen, The Netherlands. Genotyping of the *HTTLPR* polymorphism in the promoter region of *SLC6A4* (*5-HTT,* *SERT*) gene was performed by simple sequence length analysis. Call rate was 91.6%. A custom-made TaqMan assay (Applied Biosystems) was utilized to genotype the single nucleotide substitution (A–G), which is present in the *HTTLPR* long (l) allele (rs25531). Call rate was 96.5%. Concordance between DNA replicates showed an accuracy of 100%. All *lg* alleles were recoded into S, because it has been shown that this polymorphism represents low serotonin expression comparable to the S allele [37], while *la* was recoded as L. Based on these alleles, we will refer to the functionality of the expressed transporter; low (SS), intermediate (LS), and high (LL), in line with previous studies [38].

The 48 bp direct repeat polymorphism in exon 3 of *DRD4* was genotyped on the Illumina BeadStation 500 platform (Illumina.). Three percent blanks and duplicates between plates were taken along as quality controls during genotyping. Determination of the length of the alleles was performed by direct analysis on an automated capillary sequencer (ABI3730, Applied Biosystems, Nieuwerkerk a/d IJssel, The Netherlands) using standard conditions. Call rate for *DRD4* was 99.4%.

The 30 bp variable number of tandem repeat polymorphism (called *MAOA-LPR* or *MAOA-uVNTR*) was also genotyped on the Illumina BeadStation 500 platform. Three percent blanks as well as duplicates between plates were taken along as quality controls during genotyping. Call rate was 100% for *MAOA*. All polymorphisms were well within Hardy–Weinberg equilibrium (HWE; *p* values ranged from 0.77 to 0.87).

#### Genotype model

Based on previous G × E research examining differential susceptibility [23], we considered the 7-repeat of *DRD4*, the low expression of *5-HTTLPR*, and the low-activity alleles of *MAOA* as plasticity alleles for our G × E analyses. We used dominant models for *DRD4* and an additive model for the tri-allelic classification of *5-HTTLPR.* The functional status of heterozygous females is uncertain given that *MAOA* is X-linked. Based on previous findings [39], heterozygous females carrying at least one long allele (3.5, 4, or 5 repeats) were categorized in the high transcription group.

#### Covariates

We selected covariates based on differences between the classes (see Table 2): sex (0 = female, 1 = male), age at T1 in years, SES (0 = high, 1 = intermediate, 2 = low), Dutch ancestry (0 = both parents born in the Netherlands, 1 = at least one parent not born in the Netherlands), and ADHD medication use as reported by the parents (methylphenidate, dexamphetamine, and atomoxetine; 0 = no use, 1 = use at some time in the year preceding T1, T2, or T3, respectively). For G × E analyses, we also included two genetic principal component analysis scores to correct for genetic population stratification. SES was based on five indicators (professional occupation and educational attainment for both father and mother, and household income), and thereafter divided into three groups: low (< 25%), medium (25–75%), and high (> 75%). As ADHD is intricately linked with comorbid psychiatric symptoms, we did not covary for comorbidities.

### Statistical analyses

Descriptive statistics and inter-correlations among variables were examined using SPSS for Windows (Version 24.0). Due to the relatively low number of missing data (ranging from 0 to 2.3%) and to avoid the limitation of Multiple Imputation which does not give pooled results of the omnibus test, we performed the Expectation Maximization algorithm in SPSS, which imputes values based on other available variables.

#### ADHD symptom trajectories

We conducted Growth Mixture Modeling (GMM) analyses using M*plus* Version 6.12 [40] to determine groups with distinct longitudinal trajectories (i.e., latent classes). GMM analyses identify multiple classes with distinct developmental trajectories, while allowing for within-group heterogeneity in initial ADHD symptom levels and longitudinal change in ADHD symptoms, which facilitates a realistic representation of complex data [41]. In other words, GMM is a method for identifying multiple unobserved subpopulations (without the need of prespecifying the number of classes) that explores qualitative differences in growth trajectories. To account for the non-normal distribution of the ADHD symptoms, we used the MLR estimator [40]. To decide upon the optimal number of latent classes, we used the Bayesian Information Criterion (BIC; [42]), and the Lo-Mendell-Rubin adjusted Likelihood Ratio Test (aLRT; [43]). For the BIC, a lower value represents a better fitting model, taking into account increased model complexity. A significant aLRT test result indicates that a model with *k* classes is better than a model with *k*-1 classes. In addition, we evaluated entropy (correct classification rate) with values approaching 1 indicating a clear separation between the latent classes. In addition to these fit indices as a guide to identify the number of classes, we also considered the theoretical meaning of the classes.

#### Perceived parenting styles predicting class membership in ADHD symptom trajectories by parenting styles and plasticity genes

First, as a descriptive analysis of non-interest (given earlier reports in the same sample, [44]), we used *χ*^2^ tests to compare genetic variants in relation to class membership. Next, we performed separate multinomial logistic regression analyses in SPSS to investigate whether perceived parenting (i.e., rejection, overprotection, and emotional warmth) as well as their interactions with the three plasticity genes (i.e., *DRD4,* *MAOA,* and *5-HTTLPR*) predicted adolescent’s class membership in ADHD symptom trajectories. We first investigated main effects of all perceived parenting styles in one model given their inter-correlations, adjusting for aforementioned covariates. We subsequently added their G × E’s per plasticity gene in three separate models on ADHD symptom trajectories, including the covariates and all covariates × G and covariates × E interaction terms in the G × E models [45]. We report the *χ*^2^ values based on likelihood ratio tests, which indicate model fit of the overall model for each single predictor (the *χ*^2^ statistic is the difference in − 2 log-likelihoods between the final model and a reduced model that omits the effect of one predictor from the final model). We provide parameter estimates (betas, standard errors, and odds ratios, including 95% confidence intervals) of the included predictors while pairwise comparing all classes with each other. The significance level corrected for multiple comparisons regarding the overall effect (i.e., likelihood ratio tests) of our predictors of interest was *p* < 0.004 (0.05/12, i.e., three main parenting factors plus 3 × 3 G × E’s); for the remaining analyses we used a *p* value of 0.05.

#### Sensitivity analyses

We conducted a number of sensitivity analyses. First, while using multinomial logistic regression eased multivariate analyses (allowing us to specify *χ*^2^ model fit for each parenting factor in the overall multivariate model and dealing with a large number of interaction terms and covariates) as compared to testing prediction models within GMM, uncertainties regarding class membership were not accounted for in the main analyses. Therefore, we repeated the multinomial logistic regression analyses using weighted variables accounting for the probability (uncertainty) of each case being assigned to a particular class. Second, we repeated analyses separately for the three different perceived parenting styles to explore the individual contribution of each and to check whether, for example, associations of positive parenting with class membership may have been overruled by those of negative parenting, thus not reaching significance in a multivariate model. Finally, we repeated analyses using data without missing data imputation based on Expectation Maximation.

## Results

### ADHD symptom trajectories

A four-class solution of ADHD symptoms across adolescence (not including covariates) fitted best to theoretical assumptions representing interpretable trajectories and the data (Loglikelihood = − 1580.40, BIC = 3287.54; aLRT = 71.47, *p* = 0.02; entropy 0.748) as the BIC was smaller than that of the two-class solution (Loglikelihood = − 1696.90, BIC = 3475.82; aLRT = 307.78, *p* < 0.001; entropy 0.803) and three-class solution (Loglikelihood = -1617.72, BIC = 3339.83; aLRT = 151.58, *p* < 0.001; entropy 0.795). A five-class model did not provide model fit, i.e. did not converge without adjusting the model by constraining variances, therefore it was not possible to compare it with the four-class solution.

**Fig. 1:**
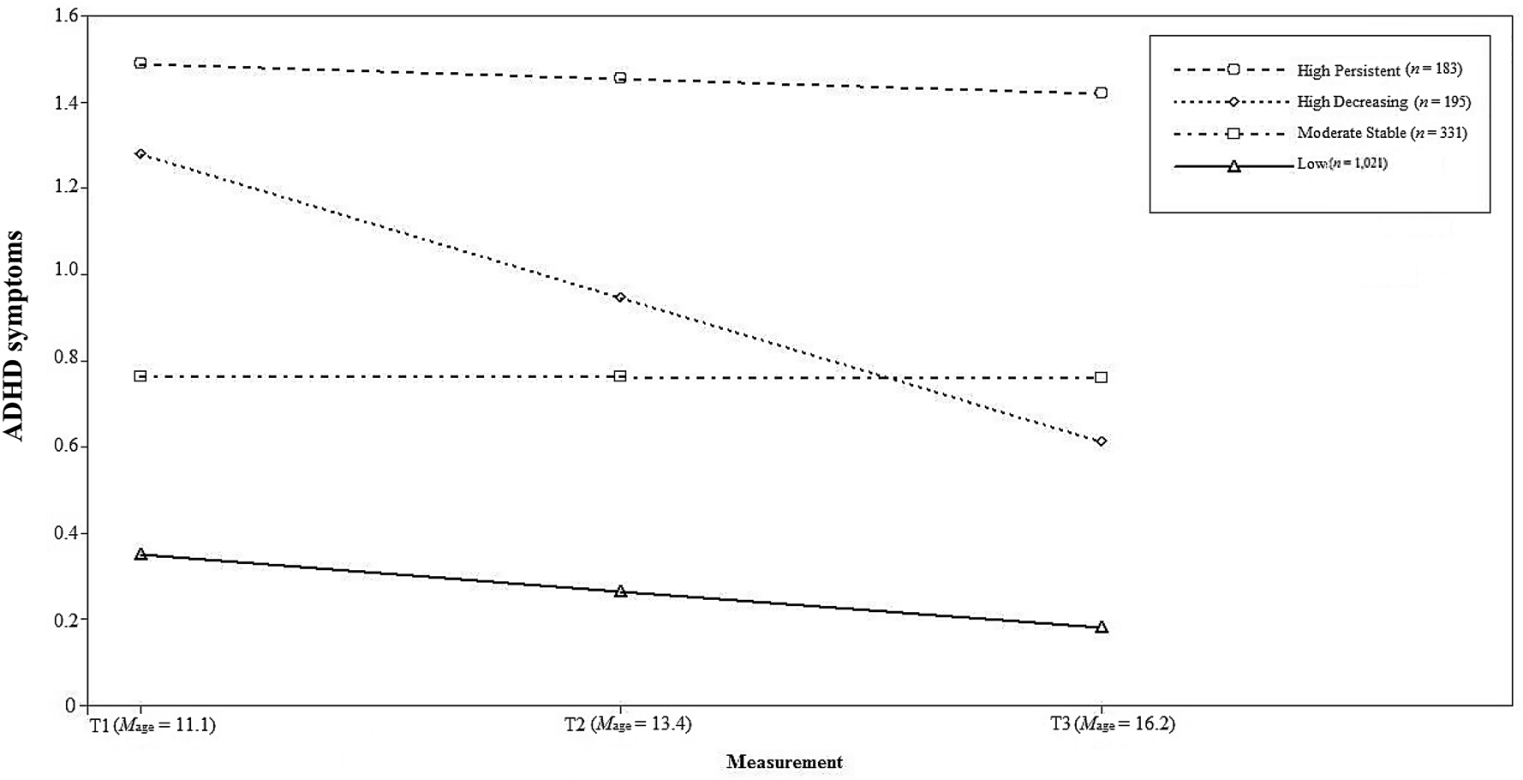
Graphical presentation of the estimated trajectories of attention-deficit/hyperactivity disorder (ADHD) symptoms from early (T1) to late (T3) adolescence using growth mixture modeling analyses, adjusted for sex, Dutch ancestry, socio-economic status, and ADHD medication use

The ADHD symptom trajectories are shown in Fig. 1. The *low* class consisted of participants with low ADHD symptoms throughout adolescence (59.0%, *n* = 1021; Intercept: *M* = 0.35, *SE* = 0.02, *p* < 0.001; Linear Slope: *M* = − 0.08, *SE* = 0.01, *p* < 0.001). The moderate stable class showed stable, moderately severe ADHD symptoms across adolescence (19.1%, *n* = 331; Intercept: *M* = 0.76, *SE* = 0.08, *p* < 0.001; Linear Slope: *M* = − 0.001, *SE* = 0.04, *p* = 0.97). Two classes presented with high initial levels of ADHD symptoms; one with high decreasing levels (10.6%, *n* = 195; Intercept: *M* = 1.28, *SE* = 0.90, *p* < 0.001; Linear Slope: *M* = − 0.33, *SE* = 0.05, *p* < 0.001), and one with stable, high persistent (11.3%, *n* = 183; Intercept: *M* = 1.49, *SE* = 0.04, *p* < 0.001; Linear Slope: *M* = − 0.04, *SE* = 0.02, *p* = 0.13) levels across adolescence.

### Sample characteristics

Correlations between study variables in the total study sample are presented in Table 1. Table 2 shows significant differences between the ADHD symptom trajectories in the main study variables and the covariates age, sex, Dutch ancestry, SES, and ADHD medication use between members of the various ADHD symptom trajectories. Table 3 shows that the *MAOA* genotype differed between the classes, i.e., the low-activity *MAOA* variant was more prevalent in the high decreasing and high persistent classes compared to the other two classes.

**Table 1:** Descriptive statistics and inter-correlations between study variables in the total sample (*n* = 1730)

Variable	1	2	3	4	5	6	7	8	9	10	11	12	13
1. Age T1	–												
2. Sex^a^	0.03	–											
3. Dutch ancestry^a,b^	− 0.04	0.02	–										
4. SES^a,c^	− 0.03	− 0.02	− 0.05*	–									
5. ADHD medication^a,d^	0.02	0.16**	0.07**	0.08**	–								
6. Rejection^e^	− 0.03	0.14**	0.03	0.06*	0.13**	–							
7. Overprotection^e^	− 0.02	0.05*	− 0.11**	0.08**	0.09**	0.44**	–						
8. Emotional warmth^e^	0.02	− 0.13**	− 0.02	− 0.12**	− 0.04	− 0.33**	0.19**	–					
9. *DRD4*^f^	0.01	0.05*	− 0.01	0.01	− 0.03	− 0.02	− 0.02	0.02	–				
10. *5-HTTLPR*^f^	0.01	− 0.03	0.05	− 0.02	− 0.03	− 0.02	− 0.02	− 0.01	− 0.01	–			
11. *MAOA*^f^	− 0.02	− 0.24**	0.004	0.02	− 0.03	− 0.02	− 0.04	− 0.01	− 0.03	0.004	–		
12. ADHD symptom severity^g^ T1	0.01	0.22**	0.01	0.23**	0.41**	0.24**	0.12**	− 0.15**	− 0.01	− 0.04	− 0.09**	–	
13. ADHD symptom severity^g^ T2	0.02	0.20**	0.04	0.19**	0.44**	0.22**	0.10**	− 0.15**	0.002	− 0.04	0.08**	0.81**	–
14. ADHD symptom severity^g^ T3	− 0.002	0.20**	0.06*	0.16**	0.46**	0.22**	0.11**	− 0.13**	− 0.02	− 0.04	− 0.07**	0.72**	0.78**

*ADHD* Attention-Deficit/Hyperactivity Disorder symptoms. *SES* socio-economic status, *DRD4* dopamine D4 receptor, *5-HTTLPR* serotonin, transporter, *MAOA* monoamine oxidase A

*p < 0.05

**p < 0.001

^a^The zero-coded categories (i.e., females, adolescents from Dutch ancestry, high socio-economic status, non-users of ADHD medication) were used as a reference group

^b^Both parents were born in the Netherlands

^c^Groups based on sum score of five indicators (household income and both parents’ occupation and education; score range 0–2)

^d^Methylphenidate, dexamphetamine, and/or atomoxetine use (1) or non-use (0) during (part of) the year before the measurement of T1, T2, and/or T3

^e^Egna Minnen Betraffande Uppfostran (EMBU-C; Markus et al. [34]; Veenstra et al. [35]) subscale sum scores (score range 0–4), assessed at T1

^f^*DRD4* coded as absence (0) or presence of 7-repeat (1); *5-HTTLPR* coded as SS carriers (0), LS carriers (1), or LL carriers (2); *MAOA* coded as low (0) or high activity (1)

^g^Mean of seven-items DSM-IV-oriented ADHD subscale of the Child Behavior Checklist (CBCL; Achenbach [31]) sum scores (score range 0–2)

**Table 2:** Sample characteristics of *n* = 1730 participants split by class membership of ADHD symptom trajectories

		Low	Moderate stable	High decreasing	High persistent	Test statistic	*p* value
		*n* = 1021 (59.0%)	*n* = 331 (19.1%)	*n* = 195 (11.3%)	*n* = 183 (10.6%)		
Population-based cohort, *n* (%)		937 (91.7%)^a^	248 (74.9%)^a^	120 (61.2%)^a^	59 (32.2%)^a^	*χ*^2^ (3) = 378.65	< 0.001
Covariates							
Age in years, *M* (*SD*)	T1	11.08 (0.56)	11.08 (0.51)	11.17 (0.56)	11.10 (0.50)	*f* (3) = 1.49	0.22
	T2	13.45 (0.57)^b,c,d^	13.33 (0.63)^b^	13.30 (0.63)^c^	13.08 (0.70)^d^	*f* (3) = 22.68	< 0.001
	T3	16.18 (0.65)	16.12 (0.70)	16.25 (0.76)^e^	16.05 (0.75)^e^	*f* (3) = 3.04	0.03
Male sex, *n* (%)		433 (42.4%)^f,g,h^	194 (58.6%)^f,i^	123 (63.1%)^g,j^	132 (72.1%)^h,i,j^	*χ*^2^ (3) = 81.90	< 0.001
Dutch ancestry, *n* (%)		929 (91%)^k^	300 (90.6%)^l^	176 (90.3%)^m^	178 (97.3%)^k,l,m^	*χ*^2^ (3) = 8.88	0.03
Socio-economic status, *n* (%)	High	360 (35.3%)^n,o,p^	83 (25.1%)^n,q,r^	32 (16.4%)^o,s^	30 (16.4%)^r,s^		
	Medium	490 (48.0%)^t,u,v^	168 (50.8%)^t,w^	103 (52.8%)^u,x^	110 (60.1%)^v,w,x^	*χ*^2^ (6) = 60.87	< 0.001
	Low	171 (16.7%)^y,z,A^	80 (24.2%)^y^	60 (30.8%)^z,B^	43 (23.5%)^A,B^		
ADHD medication use, *n* (%)		74 (7.2%)^a^	72 (21.8%)^a^	63 (32.3%)^a^	117 (63.9%)^a^	*χ*^2^ (3) = 358.01	< 0.001
ADHD symptoms, *M* (SD)							
ADHD symptom severity	T1	0.36 (0.30)^a^	0.77 (0.28)^a^	1.36 (0.27)^a^	1.52 (0.32)^a^	*f* (3) = 1214.54	< 0.001
	T2	0.22 (0.23)^a^	0.73 (0.28)^a^	0.96 (0.31)^a^	1.44 (0.32)^a^	*f* (3) = 1385.39	< 0.001
	T3	0.18 (0.68)^a^	0.80 (0.19)^a^	0.63 (0.21)^a^	1.43 (0.25)^a^	*f* (3) = 2187.33	< 0.001
Inattention symptom severity	T1	0.43 (0.41)^ C,D,E^	0.89 (0.44)^ C,F,G^	1.48 (0.37)^D,G^	1.56 (0.44)^E,F^	*f* (3) = 613.72	< 0.001
	T2	0.30 (0.37)^a^	0.95 (0.44)^a^	1.13 (0.43)^a^	1.53 (0.42)^a^	*f* (3) = 403.93	< 0.001
	T3	0.28 (0.33)^a^	1.09 (0.39)^a^	0.88 (0.34)^a^	1.59 (0.38)^a^	*f* (3) = 361.83	< 0.001
Hyperactivity/impulsivity symptom severity	T1	0.30 (0.32)^a^	0.67 (0.36)^a^	1.26 (0.38)^a^	1.48 (0.40)^a^	*f* (3) = 429.24	< 0.001
	T2	0.16 (0.24)^a^	0.56 (0.34)^a^	0.83 (0.38)^a^	1.36 (0.39)^a^	*f* (3) = 375.64	< 0.001
	T3	0.12 (0.19)^a^	0.57 (0.29)^a^	0.44 (0.29)^a^	1.30 (0.38)^a^	*f* (3) = 326.50	< 0.001
Perceived parenting, *M* (SD)							
Rejection	T1	1.46 (0.28)^H,I,J^	1.54 (0.32)^H,K^	1.56 (0.34)^I,L^	1.66 (0.37)^J,K,L^	*f* (3) = 28.35	< 0.001
Overprotection	T1	1.83 (0.36)^M,N^	1.88 (0.39)	1.95 (0.42)^M^	1.94 (0.39)^N^	*f* (3) = 8.59	< 0.001
Emotional warmth	T1	3.27 (0.47)^O,P,Q^	3.16 (0.53)^O^	3.15 (0.50)^P^	3.09 (0.54)^Q^	*f* (3) = 10.12	< 0.001

See Table 1 for abbreviations and explanation of variables

^a^Significant differences between all class memberships at *p* < 0.05

^b^^−^^L^Values by the same letter indicates a significant difference between class memberships at *p* < 0.05

**Table 3:** Comparison of genotypes in n = 1,730 participants in relation to class membership of ADHD symptom trajectories by *χ*^2^ analysis

	Low	Moderate stable	High decreasing	High persistent	Test statistic	*p* value
	*n* = 1021 (59.0%)	*n* = 331 (19.1%)	*n* = 195 (11.3%)	*n* = 183 (10.6%)		
*DRD4*^a^ *n* (%)					*χ*^2^ (3) = 1.31	0.73
7^−^ carriers	641 (62.8%)	217 (65.6%)	119 (61.0%)	117 (63.9%)		
7^+^ carriers	80 (37.2%)	114 (34.4%)	76 (39.0%)	66 (36.1%)		
*5-HTTLPR*^b^ *n* (%)					*χ*^2^ (6) = 8.06	0.23
SS	255 (25.0%)	72 (21.8%)	53 (27.2%)	59 (32.2%)		
LS	508 (49.8%)	170 (51.3%)	94 (48.2%)	87 (47.5%)		
LL	258 (25.2%)	89 (27.9%)	48 (24.6%)	37 (20.2%)		
*MAOA*^c^ *n* (%)					*χ*^2^ (3) = 19.63	< 0.001
Low activity	228 (22.3%)	81 (24.4%)	65 (33.3%)	63 (34.4%)		
High activity	793 (77.7%)	250 (75.6%)	130 (66.7%)	120 (65.6%)		

See Table 1 for abbreviations of included genes

^a^*DRD4* coded as absence (0) or presence 7-repeat (1)

^b^*5-HTTLPR* coded as SS carriers (0), LS carriers (1), or LL carriers (2)

^c^*MAOA* coded as low (0) or high activity (1)

At T1, 316 (18.3%) adolescents of the total sample (*n* = 1730) had clinical (*n* = 180, 10.4%) or subclinical (*n* = 136, 7.9%) ADHD symptom levels, based on the respective ASEBA cut-off values [33]. Of these 316 adolescents, 144 (10.6%) and 172 (47.0%) individuals were from the population-based (*n* = 1364) and clinic-referred cohort (*n* = 366), respectively.

In the high persistent ADHD symptom trajectory, respectively 109 (61.2%) of the 183 adolescents were in the clinical range at T1 and 75 (47.8%) at T3; and 42 (23.6%) at T1 and 75 (47.8%) at T3 in the subclinical range. Likewise, in the high decreasing trajectory, respectively, 67 (33.7%) of the adolescents were in the clinical range at T1 and 0% at T3, and in the subclinical range 74 (38.9%) at T1 and 8 (5.2%) at T3.

Our data further indicate that 4.3% (i.e., 59 out of 1,364) of the adolescents from the population cohort and 33.9% (i.e., 124 out of 366) adolescents from the clinic-referred cohort were following a high persistent trajectory (Table 2). Of note, the patterns of inattentive and hyperactive-impulsive symptoms were similar at T1, T2, and T3 (see Table 2).

### Perceived parenting styles predicting class membership in ADHD symptom trajectories

In the overall model including all three parenting factors simultaneously, model fit (*χ*^2^) was significant for perceived parental rejection as a predictor differentiating between ADHD symptom trajectories [*χ*^2^ (3) = 15.28, *p* = 0.002]. Model fit indices of perceived parental emotional warmth [*χ*^2^ (3) = 7.07, *p* = 0.07] and perceived parental overprotection [*χ*^2^ (3) = 5.09, *p* = 0.17] were not significant. Subsequent pairwise class comparisons (see Table 4) showed that adolescents who perceived more parental rejection at baseline during early adolescence had substantially higher odds (ORs from 2.14 to 3.74) to be a member of the high persistent trajectory across adolescence compared to adolescents in the low, moderate stable, or high decreasing classes. Also, adolescents in the moderate stable class perceived more parental rejection than in the low ADHD symptom trajectory (OR 1.75).

**Table 4:** Parameter estimates and odds ratios for perceived parenting styles as predictors of class membership in ADHD symptom trajectories

	Low^a^ vs. moderate stable		Low^a^ vs. high decreasing		Low^a^ vs. high persistent		Moderate stable^a^ vs. high decreasing		Moderate stable^a^ vs. high persistent		High decreasing^a^ vs. high persistent	
	B (SE)	OR (95% CI)	B (SE)	OR (95% CI)	B (SE)	OR (95% CI)	B (SE)	OR (95% CI)	B (SE)	OR (95% CI)	B (SE)	OR (95% CI)
Rejection	**0.56** (0.27)*,^**b1**^	**1.75** **(1.04‒2.94)**	0.45 (0.32)^**b2**^	1.57 (0.84‒2.95)	**1.32** (0.35)**,^**b1**^	**3.74** **(1.89‒7.42)**	‒ 0.11 (0.35)^b1^	0.90 (0.45‒1.79)	**0.76** (0.37)*, ^**b1**^	**2.14** **(1.05‒4.38)**	**0.87** (0.39)*, ^**b3**^	**2.38** **(1.10‒5.14)**
Over- protection	0.11 (0.21)	1.11 (0.74‒1.69)	0.58 (0.26)*	1.78 (1.08‒2.95)	0.12 (0.30)	1.13 (0.63‒2.02)	0.47 (0.29)	1.60 (0.92‒2.80)	0.02 (0.31)	1.02 (0.55‒1.88)	− 0.46 (0.33)	0.63 (0.33‒1.22)
Emotional warmth^c^	− 0.26 (0.15)^c^	0.77 (0.57‒1.04)	− 0.39 (0.19)*^,c^	0.67 (0.46‒0.98)	− 0.40 (0.21)^c^	0.67 (0.44‒1.01)	− 0.13 (0.21)	0.88 (0.58‒1.32)	− 0.14 (0.22)	0.87 (0.56‒1.35)	− 0.008 (0.24)	0.99 (0.62‒1.60)
Sex^d^	0.55 (0.13)**	1.74 (1.34‒2.26)	0.71 (0.17)**	2.04 (1.46‒2.84)	0.94 (0.20)**	2.57 (1.74‒3.79)	0.16 (0.19)	1.17 (0.81‒1.70)	0.39 (0.21)	1.48 (0.97‒2.24)	0.23 (0.23)	1.26 (0.80‒1.98)
Age^d^	0.04 (0.12)	1.01 (0.82‒1.32)	0.36 (0.15)*	1.44 (1.07‒1.93)	0.17 (0.18)	1.18 (0.34‒1.67)	0.32 (0.17)	1.38 (0.99‒1.92)	0.13 (0.19)	1.13 (0.79‒1.64)	− 0.20 (0.20)	0.82 (0.56‒1.22)
SES^d^												
Low	0.67 (0.19)**	1.96 (1.35‒2.85)	1.13 (0.25)**	3.72 (2.28‒6.07)	1.04 (0.29)**	2.83 (1.60‒5.00)	0.64 (0.28)*	1.90 (1.11‒3.25)	0.37 (0.30)	1.44 (0.80‒2.62)	− 0.27 (0.34)	0.76 (0.39‒1.47)
Medium	0.42 (0.16)*	1.52 (1.12‒2.06)	0.91 (0.22)**	2.48 (1.60‒3.83)	1.0 (0.25)**	2.86 (1.77‒4.62)	0.49 (0.25)*	1.63 (1.01‒2.64)	0.63 (0.26)*	1.89 (1.13‒3.14)	0.14 (0.30)	1.16 (0.64‒2.07)
Dutch ancestry	− 0.09 (0.23)	0.92 (0.59‒1.43)	− 0.03 (0.29)	0.97 (0.55‒1.69)	0.92 (0.50)	2.57 (0.96‒6.86)	0.05 (0.32)	1.06 (0.57‒1.97)	1.03 (0.52)*	2.80 (1.02‒7.70)	0.98 (0.53)	2.65 (0.94‒7.54)
ADHD medication	1.19 (0.18)**	3.27 (2.28‒4.69)	1.68 (0.20)**	5.36 (3.60‒7.98)	2.96 (0.21)**	19.22 (12.86‒28.74)	0.49 (0.21)*	1.64 (1.09‒2.46)	1.78 (0.21)**	5.88 (3.91‒8.84)	1.28 (0.22)**	3.58 (2.32‒5.54)

Statistically significant differences between pairs of classes of ADHD symptom trajectories shown at *p* < 0.05. Bold statistics refer to the preceding significant overall effect at *p* < 0.004 corrected for multiple testing. Betas (B), standard error (SE) and odd ratios (OR) and 95% confidence intervals (CI) from multivariate multinominal logistic regression. A higher OR (> 1) indicates that a higher perceived parenting score is associated with a higher risk to be a member of the higher order trajectory, whereas a lower OR (< 1) is associated with a lower risk. Nagelkerke *R*^2^ = 0.27

*p < 0.05

**p < 0.001 presents significant within-group differences

^a^Reference group

^b^Sensitivity analyses using weighted variables taking the probability of belonging to a class into account

^b1^Main result confirmed

^b2^Became significant

^b3^Lost significance; see main text for statistics

^c^Note that emotional warmth retained significant effects in a model without including parental rejection and overprotection. For results, see the main text

^d^The zero-coded categories (i.e., females, adolescents from Dutch ancestry, high socio-economic status, non-users of ADHD medication) were used as a reference group

#### Sensitivity analyses

The main results were confirmed when using weighted variables taking the probability of class membership into account (Table 4). Parental rejection also emerged as a significant predictor in the overall model [*χ*^2^ (3) = 17.02, *p* = 0.001], whereas, again, parental emotional warmth [*χ*^2^ (3) = 6.02, *p* = 0.11] and overprotection [*χ*^2^ (3) = 4.05, *p* = 0.257] were not significant in the overall model. Pairwise comparisons between classes of ADHD symptom trajectories indicated higher odds of adolescents perceiving more parental rejection in the high persistent class (*B* = 1.36, SE = 0.35, OR 3.9, 95% CI 1.67‒7.7), *p* = 0.001), the high decreasing class (*B* = 0.70, SE = 0.35, OR 2.01, 95% CI 1.02‒3.9), *p* = 0.043), and in the moderate stable class (*B* = 0.64, SE = 0.28, OR 1.89, 95% CI 1.09‒3.28, *p* = 0.024) compared to the low class; and of those in the high persistent class compared to the moderate stable class (*B* = 0.73, SE = 0.38, OR 2.07, 95% CI 1.0‒4.30 *p* = 0.05). While, again, there was no difference between the moderate stable and high decreasing class regarding perceived parental rejection (*B* = 0.06, SE = 0.38, OR 1.06, 95% CI 0.50‒2.26, *p* = 0.87), the difference between the high decreasing and high persistent class was no longer significant (B = 0.66, SE = 0.41, OR 1.94, 95% CI 0.88‒2.26, *p* = 0.10).

Furthermore, the sensitivity analyses with a sole parenting predictor of ADHD symptom trajectories not adjusting for the other two parenting variables showed that perceived parental rejection was still significant [*χ*^2^ (3) = 38.59, *p* < 0.001], and thus unaffected by the other two parenting factors, whereas perceived parental overprotection remained not significant [*χ*^2^ (3) = 3.85, *p* = 0.28]. The model without perceived parental rejection and overprotection now showed a significant effect for emotional warmth [*χ*^2^ (3) = 16.79, *p* < 0.001]. Adolescents in the high persistent class [*B* = − 0.65, SE = 0.18, OR 0.52, 95% CI 0.37‒0.74, *p* < 0.001], high decreasing class [*B* = − 0.37, SE = 0.16, OR 0.69, 95% CI 0.50‒0.95 *p* = 0.02], and moderate stable class [*B* = -− 0.35, SE = 0.13, OR 0.71, 95% CI 0.55‒0.91, *p* = 0.008] perceived less emotional warmth from their parents than those in the low class. Results thus suggest an opposite pattern for perceived emotional warmth, predicting membership in the low trajectory, compared to parental rejection which was related to a high persistent trajectory. Finally, sensitivity analyses using data without missing data imputation with Expectation Maximation did not show a notable change of results.

### Plasticity genes × perceived parenting styles predicting class membership in ADHD symptom trajectories

There were no significant interactions between the *DRD4* genotypes and perceived parental rejection [*χ*^2^ (3) = 2.70, *p* = 0.44], overprotection [*χ*^2^ (3) = 1.05, *p* = 0.79], and emotional warmth [*χ*^2^ (3) = 1.80, *p* = 0.62] in predicting class membership in ADHD symptom trajectories. Also, no significant interactions were found between the *5-HTTLPR* genotype and perceived parental rejection [*χ*^2^ (6) = 2.06, *p* = 0.92], overprotection [*χ*^2^ (6) = 2.55, *p* = 0.86], and emotional warmth [*χ*^2^ (6) = 6.56, *p* = 0.36], nor between the *MAOA* genotype and perceived parental rejection [*χ*^2^ (3) = 1.73, *p* = 0.63], overprotection [*χ*^2^ (3) = 0.57, *p* = 0.90], and emotional warmth [*χ*^2^ (3) = 3.19, *p* = 0.36]. Although variables of non-interest, the main effects of the *DRD4,* *5-HTTLPR,* *and* *MAOA* genotypes in differentiating between the ADHD symptom trajectories were also not significant in the multivariate analyses. In contrast to the univariate analysis (see Table 3), the effect of the *MAOA* genotype became non-significant (*p* values > 0.05) after correcting for sex, and in a post hoc analysis conducting analyses separately for boys and girls, which is important as the *MAOA* genotype is linked to the X chromosome.

## Discussion

In the present study, we examined perceived parental rejection, overprotection, and emotional warmth, as well as their interaction with three plasticity genes as predictors of class membership in ADHD symptom trajectories across adolescence (age range 10–18 years) in a large pooled population and clinic-referred sample. We identified four different ADHD symptom trajectories across adolescence: low, moderate stable, high decreasing, and high persistent. While G × E’s with three different plasticity genes did not predict class membership in ADHD symptom trajectories, perceived parental rejection discriminated between the high persistent and the other three classes. More specifically, adolescents following the high persistent ADHD symptom trajectory perceived more parental rejection during early adolescence than adolescents in the other trajectories. In contrast, higher perceived parental emotional warmth was linked to the low ADHD symptom trajectory, suggesting a protective role, at least in a model without the other two parenting styles. Finally, the association of the low activity *MAOA* variant with the high decreasing and high persistent classes is in line with previous studies pointing to a role of low-activity *MAOA* in externalizing behaviors [46].

Our study points to developmentally distinct patterns of ADHD symptoms across adolescence, showing that a small group (10.6%) of youth from the general population and an additional high-risk sample is at risk of persistently high levels of ADHD symptoms. This percentage should be seen in light of our mixed sample, pointing to persistence of a high class of ADHD symptom levels in 4.3% of adolescents from the general population cohort and 33.9% of adolescents from the clinic-referred cohort, respectively. Therefore, the high persistent trajectory confers to the broader spectrum of ADHD symptoms and includes adolescents with clinical and subclinical levels of ADHD symptoms, as well as a small proportion with non-clinical symptom levels which were yet higher than those of others in the non-clinical range. Nevertheless, our findings are broadly in line with previous studies that also identified four ADHD symptom trajectories across childhood or adolescence, with 2.8–5% following a high persistent ADHD symptom trajectory in population-based samples [5, 47] and 17.5–22% in clinical samples [6, 7]. Unlike our study, trajectories with increasing symptom severity have also been reported in a variety of samples [48, 49]. This may be explained by the younger age of the study subjects in those samples, given that ADHD symptomatology typically peaks during childhood, but tends to remit mainly during adolescence [5], while recently reported late-onset ADHD typically manifests beyond 16 years [50]. In line with our study, other reports covering adolescence also did not show an increasing ADHD symptom trajectory [5, 47].

An important new contribution of this study to the existing literature is that perceived parental rejection in early adolescence is linked to risk of a trajectory of persistently high ADHD symptoms across adolescence. Our study thus points to a long-term predictive role of perceived parental rejection that exists beyond childhood, across early to a later stage of adolescence. Our finding is in line with Sasser and colleagues [14] who demonstrated that inconsistent parenting in childhood, another example of a negative parenting style, was associated with a persistently high class of clinically significant ADHD symptoms into late adolescence. It also fits with findings of negative parenting in early childhood (i.e., lower emotional support and lower intellectual stimulation), predicting class membership of consistently high levels of inattention or hyperactivity throughout adolescence [13].

Although our study does not allow for making inferences about causality of parenting, as is inherent to observational studies, some literature does suggest causality between parenting and child behavior over time. For example, a twin study found that hostile parenting behavior of adoptive mothers prospectively predicted ADHD symptoms 1.5 years later in 6-year-old children [51]. In reverse, adolescent’s ADHD and associated difficult behavior (e.g., moodiness, uncooperativeness) may place a high strain on the family system and possibly increase parental stress facilitating rejection that often occurs in families of children with ADHD [52]. Thus, parenting styles and adolescent’s own behavior may reinforce each other in a vicious cycle over time, i.e. evocative gene-environment correlation may be involved [53]. Indeed, in a longitudinal study with a 1-year time interval involving 194 school-aged children, children’s ADHD symptoms led to increased mother–child rejection, whereas paternal rejection exacerbated children’s ADHD symptoms [54]. Most likely, therefore, is the existence of bidirectional relations between ADHD symptoms and parental rejection. One possibility to explain the impact of parental rejection on the persistence of ADHD symptoms is that rejecting parents may fail to provide an emotionally supportive environment, which is a crucial aspect in parenting a child with ADHD symptoms. Furthermore, because ADHD is highly heritable, adolescents with ADHD more often have parents with ADHD which is associated with more negative parenting [55]. Clearly, more longitudinal bidirectional studies across adolescence are needed to support a causal role of parental rejection on persisting ADHD symptom trajectories. Further, more studies with sophisticated family designs (e.g., twin, sibling studies) are necessary to control for unmeasured genetic confounding, as the association between parental behaviors or the adolescent’s experience of the home environment and the adolescent’s ADHD symptoms may be (partly) explained by shared genetic factors, thus not implying causality (see e.g. [56]).

Perceived parental overprotection did not predict class membership in ADHD symptom trajectories, while emotional warmth was significant only when examined as a single parenting predictor in the model. Thus, negative parenting (i.e., rejection) seems more strongly related to ADHD symptom trajectories than positive parenting. Still, adolescents following the low trajectory of ADHD symptoms perceived more emotional warmth from their parents in early adolescence as compared to adolescents in the moderate stable, high decreasing, and high persistent trajectories who experienced lower emotional warmth. The association of parental emotional warmth in our study with a low ADHD symptom trajectory is in line with a study that showed that positive parenting was linked with good adolescent mental health over time [57]. It has been suggested that positive parenting is even more critical in the early years of children’s development [58]. Indeed, a longitudinal study in 5-to-13-year-old children found that the association between higher parental warmth and ADHD declined over a 2-year period [59]. Our focus on adolescence may explain why we did not find a promotive role of emotional warmth with regard to remission of ADHD symptoms. Finally, whereas other studies showed that overprotection was associated with higher levels of externalizing behavior [36, 60] and that adolescents with and without persisting ADHD received more maternal overprotection [9], our findings do not support a role of perceived parental overprotection in predicting ADHD symptom trajectories across adolescence. This is in line with Musser and colleagues [6] who also found no differences for any of the ADHD symptom trajectory groups ranging from 7 to 13 years of age regarding a comparable measure of overprotection (i.e., emotional over-involvement).

We did not find G × E interactions involving the *DRD4,* *MAOA*, and *5-HTTLPR* genotype on ADHD symptom trajectories across adolescence. The absence of an interaction with the *DRD4* genotype was in contrast with Berry and colleagues [25] who found that in the context of highly sensitive early maternal care, the *DRD4* 7-repeat was associated with trajectories with low levels of inattention across childhood, whereas insensitive early maternal care in children with the *DRD4* 7-repeat polymorphism was associated with high levels of inattention. Unlike our study, Berry and colleagues [25] examined to what extent parenting was “tuned” to the child’s demands in a given situation. Another explanation for the absence of G × Parenting interactions may be that childhood is a more critical period for influences of parenting than adolescence [61]. This might explain why the childhood study of Berry and colleagues [25] showed significant results and our adolescent study did not. Also, all other cross-sectional G × Parenting findings on ADHD symptoms [27, 28] were conducted in childhood with one exception [26], a study that examined the long-term effect of early maternal care at age of 3 months at ADHD symptoms in adolescence. It is possible, therefore, that especially early caregiving is of importance in examining G × E on the course of ADHD symptoms across development.

### Strengths and limitations

A strength of this study was the use of a large longitudinal dataset of adolescents, which consisted of a population sample enriched by data from a clinic-referred cohort. This enabled us to identify ADHD symptom trajectories in relation to parenting style covering the full continuum from non-clinical to clinical ADHD symptom levels across adolescence, an age range that is still underrepresented in the ADHD literature. While we focused on a measure that most closely mirrors ADHD criteria of the DSM, a potential limitation of the use of the DSM-oriented ADHD subscale is that it does not capture all 18 ADHD symptoms of the DSM; however, this subscale has been shown to have adequate diagnostic accuracy [32]. Moreover, given the low number of items, we did not define trajectories as per ADHD subdimensions (i.e., separately for inattentive and hyperactive-impulsive symptoms). Nevertheless, when comparing the mean ADHD subdimension scores across the four trajectories, the pattern for inattention and hyperactivity-impulsivity appeared largely similar, thus not suggesting profound differences between the two. We also did not use teacher ratings to assess ADHD symptoms and lack information about psychosocial treatment that some families may have received. The entropy value was below the desired threshold of 0.80 and limits confidence in the robustness of the four-class solution; therefore results need to be replicated in future studies.

Moreover, despite the EMBU’s good psychometric properties [34, 62], the assessment of adolescent’s subjective perception of parenting styles may lead to possible biases [63], compared to measurements of observed parental behavior; yet the latter are difficult to obtain in large-scale studies. Particularly in the context of ADHD, adolescent self-report of parenting behaviors may be associated with hostile attribution bias, blaming others for own mistakes or misbehaviors, or to have a parent whose negative parenting style is specifically elicited by the adolescent's behavior. Another limitation is that we measured perceived parenting only at baseline, and at a relatively late age (i.e., not already during childhood). Additionally, examining parent ratings for fathers and mother separately or the effects of parental psychopathology (including ADHD) might be relevant for future research.

Finally, the literature has raised criticism about inconsistent G × E studies with likely false positive findings [64]. However, given the large (methodological) differences between studies (e.g., in parenting variables or plasticity genes examined), it is difficult to determine whether findings are truly inconsistent or are simply incomparable [65]. Considering differences across studies, an attempt has been made to address one of these issues using polygenic risk scores [47].

## Conclusions

Our study highlights that perceived parental rejection in early adolescence is a predictor of persistently high ADHD symptoms into later adolescence. In contrast, positive parenting may have a protective role associated with low symptom levels. To address causality, future studies are needed to clarify the specific directions through which effects between parenting and adolescents’ ADHD symptoms are operating, preferably investigating a broad set of parenting variables across multiple time points. Clinical interventions, particularly parent training programs for children with ADHD, are advised to focus on the prevention of parental rejection and improvement of parenting skills in mitigating ADHD symptoms which might have beneficial long-term effects.

We could not find support for a role of the individual plasticity genes *DRD4*, *MAOA*, and 5*-HTTLPR* in interaction with parenting in the prediction of ADHD symptom trajectories. Future family-based studies that separate genetic and familial influences, as well as genetic studies utilizing polygenic risk scores or gene-sets, may be better suited to capture the contribution of genes and environment on the course of ADHD.

## Data Availability

Data can be obtained from the last author (AD).
